# Intraoral photobiomodulation-induced orthodontic tooth alignment: a preliminary study

**DOI:** 10.1186/s12903-015-0159-7

**Published:** 2016-01-13

**Authors:** Timothy Shaughnessy, Alpdogan Kantarci, Chung How Kau, Darya Skrenes, Sanjar Skrenes, Dennis Ma

**Affiliations:** Shaughnessy Orthodontics, 4330 Johns Creek Parkway, Suite 500, Suwanee, GA USA; Department of Applied Oral Health Sciences, Forsyth Institute, 245 First Street, Cambridge, MA USA; Department of Orthodontics, Faculty of Dentistry, University of Alabama, 1919 7th Ave S, SDB 305 Birmingham, AL USA; Biolux Research Ltd, 220-825 Powell St, Vancouver, B.C, Canada

**Keywords:** Orthodontic tooth movement, Intraoral photobiomodulation, Alignment, Accelerates, Treatment time, Low-level light therapy

## Abstract

**Background:**

Numerous strategies have been proposed to decrease orthodontic treatment time. Photobiomodulation (PBM) has previously been demonstrated to assist in this objective. The aim of this study was to test if intraoral PBM increases the rate of tooth alignment and reduces the time required to resolve anterior dental crowding.

**Methods:**

Nineteen orthodontic subjects with Class I or Class II malocclusion and Little’s Irregularity Index (LII) ≥ 3 mm were selected from a pool of applicants, providing 28 total arches. No cases required extraction. The test group (*N* = 11, 18 arches, 10 upper, 8 lower) received daily PBM treatment with an intraoral LED device (OrthoPulse™, Biolux Research Ltd.) during orthodontic treatment, while the control group (*N* = 8, 10 arches, 3 upper, 7 lower) received only orthodontic treatment. The PBM device exposed the buccal side of the gums to near-infrared light with a continuous 850-nm wavelength, generating an average daily energy density of 9.5 J/cm^2^. LII was measured at the start (T0) of orthodontic treatment until alignment was reached (T1, where LII ≤ 1 mm). The control group was mostly bonded with 0.018-in slot self-ligating SPEED brackets (Hespeler Orthodontics, Cambridge, ON. Canada), while conventionally-ligating Ormco Mini-Diamond twins were used on the PBM group (Ormco, Glendora, Calif. USA). Both groups progressed through alignment with NiTi arch-wires from 0.014-in through to 0.018-in (Ormco), with identical arch-wire changes. The rate of anterior alignment, in LII mm/week, and total treatment time was collected for both groups. Cox proportional hazards models were used to compare groups and while considering age, sex, ethnicity, arch and degree of crowding.

**Results:**

The mean alignment rate for the PBM group was significantly higher than that of the control group, with an LII change rate of 1.27 mm/week (SD 0.53, 95 % CI ± 0.26) versus 0.44 mm/week (SD 0.20, 95 % CI ± 0.12), respectively (*p* = 0.0002). The treatment time to alignment was significantly smaller for the PBM group, which achieved alignment in 48 days (SD 39, 95 % CI ± 39), while the control group took 104 days (SD 55, 95 % CI ±19, *p* = 0.0053) on average. These results demonstrated that intraoral PBM increased the average rate of tooth movement by 2.9-fold, resulting in a 54 % average decrease in alignment duration versus control. The average PBM compliance to daily treatments was 93 % during alignment.

**Conclusions:**

Under the limitations of this study, the findings suggest that intraoral PBM could be used to decrease anterior alignment treatment time, which could consequently decrease full orthodontic treatment time. However, due to its limitations, further research in the form of a large, randomized trial is needed.

**Trial registration:**

ClinicalTrials.gov NCT02267837. Registered 10 October 2014.

## Background

The duration of orthodontic treatment is of primary concern of patients. Depending on a patient’s malocclusion and orthodontic treatment plan, fixed appliance therapy may last 20–30 months [1, 2]. This lengthy process can be a major deterrent for many prospective orthodontic patients.

One potential way of reducing orthodontic treatment time is by increasing the rate of tooth movement. Several methods aim to achieve this through the stimulation of bone remodeling [3]. Some of these methods are the injection of vitamin D[4], prostaglandins[5], osteocalcin[6] and relaxin[7] around the alveolar socket, but more invasive techniques are also used, such as surgical injury to the cortical bone (decortication, piezocision or corticision). Although these have been found to increase tooth movement rate, they are associated with discomfort, pain and invasiveness. There is a need for truly non-invasive and user-friendly methods of reducing treatment time.

Photobiomodulation (PBM), also known as low-level light therapy (LLLT), attempts to use low energy lasers or light-emitting diodes (LED) to modify cellular biology by exposure to light in the red to near-infrared (NIR) range (600–1000 nm). NIR light therapy has been linked to increased mitochondrial metabolism[8], wound healing[9] and the promotion of angiogenesis[10] in skin, bone, muscle, and nervous tissues[11].

Clinically, the effect of NIR therapy on pain has been investigated in large number of studies, although the quality of clinical trials may be lacking[12]. There is evidence that lasers in 800–850 nm range may decrease orthodontic pain, TMJ pain and joint pain, at least in the short term[13, 14]. NIR LED therapy has shown effects in reducing muscle fatigue[15], healing bone grafts[16, 17] and the prevention of sarcopenia[18].

At the cellular level, NIR exposure is thought to activate the primary mitochondrial photoacceptor of light, cytochrome c oxidase (COX)[19, 20]. COX activation results in various cellular responses, including increased mitochondrial ATP production[21]. Increased ATP levels may accelerate bone remodeling through overall elevation of metabolic activity. LLLT may also promote angiogenesis, increasing the blood supply necessary for remodeling[22].

Preliminary data from a rat model has proposed that using low-level lasers can accelerate tooth movement. Increased osteoclast numbers were observed on the compression side of the moved molars, with an increase in bone formation and cellular proliferation on the tension side[23]. Preliminary clinical data suggests significantly faster canine retraction resulting from low-intensity laser therapy[24–26].

Recently, systematic reviews have investigated the evidence that laser therapy can accelerate tooth movement[27]. Kalemaj Z et al. concluded that there was some evidence of an effect, but that the effect size was not clinically relevant. Carvalho-Lobato et al. found statistically significant changes in four out of five human studies and eight out of eleven animal studies, and concluded that a reasonable dose of varying wavelength may reduce orthodontic treatment time[28]. However, inaccuracies in the inclusion/exclusion criteria and study selection of this review have been criticized[29]. Gkantidis et al. evaluated eighteen clinical trials and conceded that some evidence existed for the efficacy of laser therapy but the evidence was “very weak” for PBM[30]. It is clear that more research is required in this area, particularly focusing on wavelength, treatment time, and power density.

In our previous study, we found that PBM with an 850-nm extra-oral LED array increased the mean rate of tooth movement during alignment by 2.3-fold[31]. While this method showed promise, extra-oral energy delivery required significantly higher output due to the absorption of light by the soft tissue.

The present study was designed as a preliminary study to assess the feasibility and impact of an intraoral PBM device. Our null hypothesis predicted that there would be no difference in the rate of orthodontic anterior alignment between the PBM and control groups.

## Methods

### Human subjects

The study design and all patient forms, including the study consent form, received ethics approval from an independent institutional review board (BL-0609B v3 23-JULY-2010, IRB Services FL IRB, USA).

**Fig. 1:**
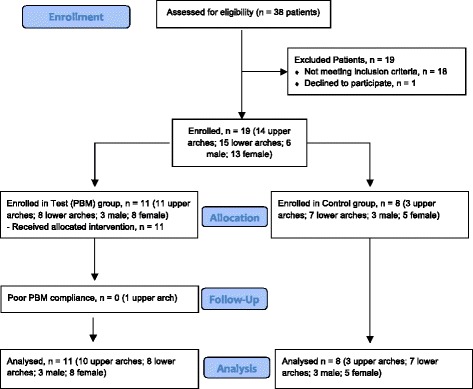
CONSORT flowchart showing patient and arch flow during the trial

Nineteen subjects (6 males, 13 females; from 11 to 18 years old) who presented for orthodontic treatment in a private practice office in Suwanee, Georgia, were recruited for the study from a pool of applicants (Fig. 1). The following inclusion criteria were used: presence of permanent dentition, eligible and scheduled for full mouth fixed orthodontic treatment, Class I or Class II malocclusion (no more than ½ cusp in Class II), non-extraction in all quadrants, non-smoker, good oral hygiene as determined by the investigator, and no adjunct treatment such as extra- or intraoral appliances. All patients had a Little’s Irregularity Index (LII)[32] greater than or equal to 3 mm. Patients were selected and treated between September 2011 and September 2013. None dropped out of the trial. Their demographics are shown in Table 1.

**Table 1:** Demographic and clinical characteristic of all patients

		Total		Control		PBM		
		mean or %	SD	mean or %	SD	mean or %	SD	*p*-value*
Demographic Characteristics								
	*n*	19		8		11		
	Age (years)	13.9	1.7	13.5	1.8	14.1	1.7	NS
	Female (%)	68.4		62.5		72.7		NS
	Caucasian (%)	68.4		75		63.6		NS
Clinical Characteristics								
	Number of Arches	28		10		18		
	Upper Arches	13		3		10		NS
	Lower Arches	15		7		8		NS
	Mandible (%)	53.6		70		44.4		NS
	Crowding (LII - mm)	6.7	2.6	5.8	1.6	7.3	3.0	NS

NS - Not significant

*p-value for comparison of group means by Mann-Whitney U or differences in proportions by chi-square test as appropriate

* *p*-value cutoff of 0.05 is used to determine significance

Beyond the possible side effects that can result from general orthodontic treatment, there are no known risks associated with the PBM device other than the unlikely chance of skin irritation from the material or the chewing and accidently swallowing of device materials. After the side effects and the benefits associated with using the device were clearly stated, each subject and his or her legal responsible approved participation in the study through signed consent.

The first 8 patients were enrolled as part of the control group, providing 10 arches eligible for the collection of alignment data (3 upper and 7 lower) as shown in Table 1. The subsequent 11 patients, of whom 7 were treated on both arches, provided a total of 18 treatment arches (10 upper and 8 lower). 3 lower arches were excluded in the PBM group for not meeting the initial dental criteria, as were 5 upper arches and 1 lower arch in the control group. The PBM group was taught how to properly use the OrthoPulse™ PBM device, and were instructed to report any adverse events immediately to the investigating orthodontist. Only 1 upper arch was excluded from the PBM group, as the patient had trouble maintaining PBM compliance for that arch.

### Device description

Test subjects used a PBM device (OrthoPulse™, Biolux Research, Vancouver, Canada), which produces near-infrared light with a continuous 850-nm peak wavelength. Patients received an average of 3.8 min of buccal-only treatment per arch per day, using an average power density of 42 mW/cm^2^ to achieve a mean energy density of approximately 9.3 J/cm^2^ at the surface of the LED array.

**Fig. 2:**
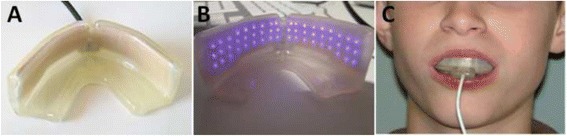
OrthoPulse™ mouthpiece. Panel **a** displays a view of the back of the device. Panel **b** showcases the LED array when the device is switched on. Panel **c** provides a view of the device *in situ*

OrthoPulse™ consists of an intraoral appliance connected to a handheld controller (Fig. 2). The controller houses the microprocessor, LCD screen and controls for the menu-driven software. It connects to a medically approved wall wart UL-2601 certified power supply (FW7555m/15, UL rating 2601, Certified IEC 60601–1). The mouthpiece is made of a flex circuit of LED arrays embedded in medical grade silicone. Light is delivered through the buccal alveolar soft tissue into the alveolus. Any heat created as a by-product of light generation was monitored and kept below the thresholds of electro-medical device safety standards.

The device recorded every full PBM treatment session completed. This provided compliance data for each patient in the treatment group.

### Orthodontic alignment of anterior teeth

Patients in the control group started orthodontic treatment before the PBM group. Due to a change in the type of brackets preferred by the clinical practice, the control group was bonded with 0.018-in slot self-ligating (SL) SPEED brackets (Hespeler Orthodontics, Cambridge, ON. Canada), but most patients in the PBM group were bonded with 0.018-in slot conventionally-ligated (CL) Ormco Mini-Diamond twin brackets (Ormco, Glendora, Calif. USA). Both groups progressed through alignment with NiTi arch-wires from 0.014-in through to 0.018-in (Ormco), with arch-wires changed in the same manner.

Complete records were obtained, including initial intraoral photographs, model casts and panoramic radiographs. Prior to bonding, the same operator (TS) collected all records. T0 defined the date of bonding and the first PBM treatment, if applicable. Intraoral photos and PBM compliance were collected at every follow-up appointment, scheduled every 3 weeks. When a patient’s LII was visually estimated to have reached ≤1 mm, T1 was recorded and a T1 model was cast. A qualified technician evaluated T1 LII, and was blinded to which patients the models originated from.

All anterior dentition crowding (LII) was measured to the nearest 0.1 mm with a fine-tip digital caliper (Point Digital Calliper SC02, Tresna Instruments, Guilin, China) by the same qualified technician. LII is the sum of the 5 linear distances from one anatomic contact point to the adjacent contact point of the 6 anterior teeth. It has been extensively used to document the degree of anterior tooth crowding, and Bernabé et al. concluded that LII is an accurate and valid method for measuring anterior arch length discrepancy[32–34]. LII measurements were made on initial models (T0) and aligned models (T1). The weekly rate of crowding resolution was calculated as:

$$ \frac{\left(\mathrm{T}1\ \mathrm{L}\mathrm{I}\mathrm{I}\ \mathrm{score}\right)\ \hbox{--}\ \left(\mathrm{T}0\ \mathrm{L}\mathrm{I}\mathrm{I}\ \mathrm{score}\right)}{\left(\mathrm{T}1\ \mathrm{date}\right)\ \hbox{--}\ \left(\mathrm{T}0\ \mathrm{date}\right)}\times 7\ \mathrm{days}/\mathrm{week} = \mathrm{rate}\ \mathrm{per}\ \mathrm{week} $$

### Statistical analysis

During study design, a sample size calculation was performed. Its parameters were based on conservative assumptions and the findings from our previous study[31]. A mean treatment effect of 2 times control alignment rate and a standard deviation of 50 % of the mean were assumed. The power analysis used a two-tailed alpha of 0.05 and statistical power of 0.80 - commonly accepted cut-offs. The analysis indicated that a minimum of 8 arches in each group was sufficient for the study to be clinically significant at the given effect size.

A variety of different tests were conducted to assess differences between treatment groups. Continuous variables (initial crowding and age) were evaluated for normalcy visually and via a Shapiro-Wilks test. Both variables were insufficiently normal, so the nonparametric Mann–Whitney *U* test was used to assess group differences. The chi-square test was used for categorical variables (sex, ethnicity, and arch). Although the difference in the number of arch type (upper vs. lower) provided in each group appears to be large, it was not found to be statistically significant (*p* = 0.194). However, the group sizes are small, decreasing the accuracy of chi-square results. Because of this, we included arch as a possible predictor in a set of post-hoc Cox proportional hazards models.

After identifying any demographic and clinical differences, a log-rank test for equality of the survivor functions was used to evaluate any difference between the control and PBM groups. This test is appropriate to the nature of our study (two-samples with right skewed distributions measured in the form of time-to-event data).

Finally, the post-hoc Cox proportional hazards models were prepared to compare crowding resolution rate ratios for treatment type while controlling for demographic and clinical variables.

To assess intra-examiner reliability of LII measurement, 8 models were randomly selected from the entire model pool to be re-measured three months before the last patients completed alignment. Pearson’s Interclass Correlation Coefficient (ICC) and a Mann–Whitney U were used to assess the accuracy and reproducibility of the LII method. Reliability analysis was completed following study completion.

All analyses were performed with the *Stata 12* statistical package (StataCorp, College Station, Texas).

## Results

The final sample consisted of 18 PBM-treated arches and 10 control arches, all non-extraction with T0 LII ≥ 3.0 mm (Table 1). All significance levels were assessed using a p-value cut-off of 0.05. There were no significant differences in age, sex or ethnicity between the two groups, nor in the ratio of mandible to maxillary arches and initial crowding.

**Fig. 3:**
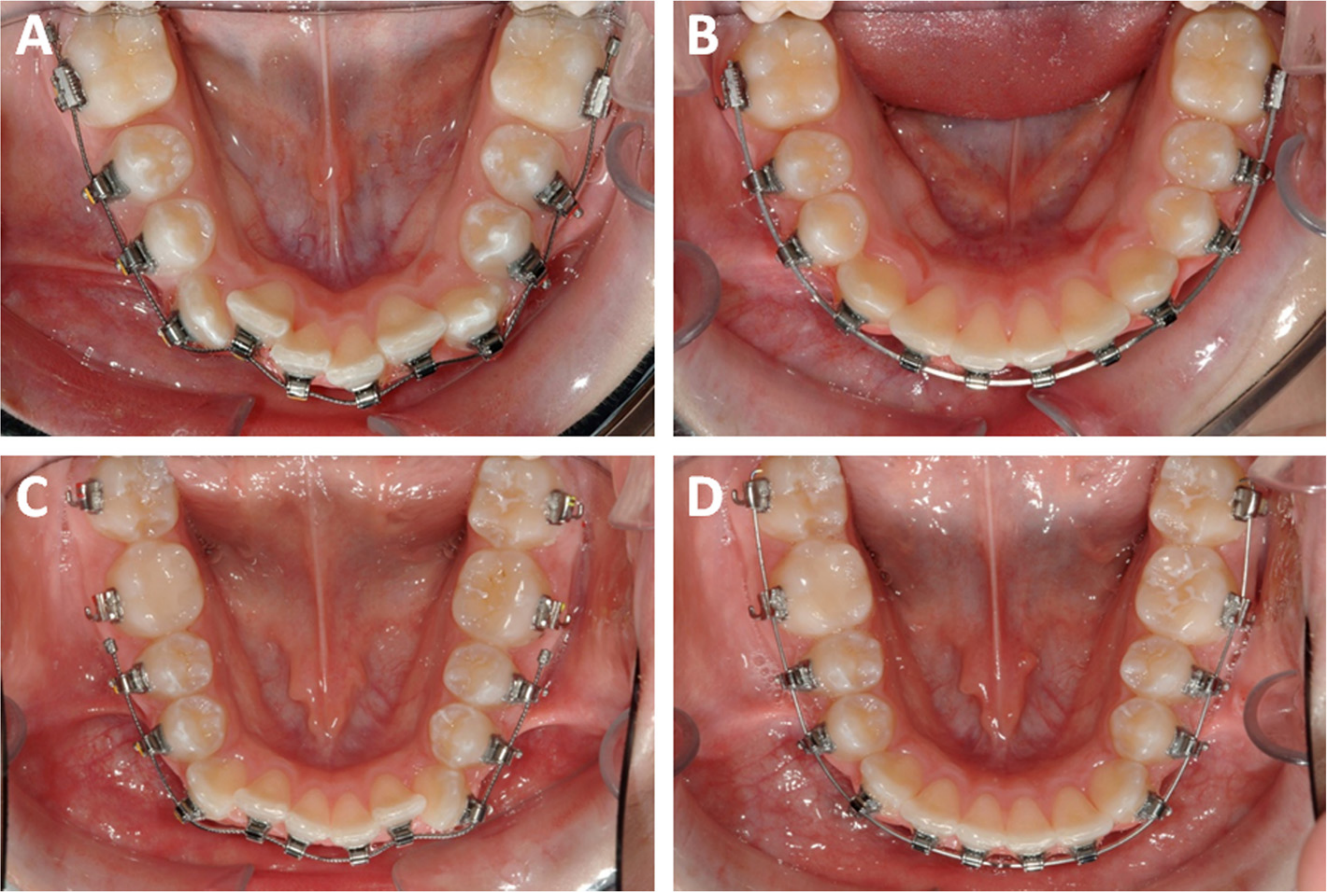
Two representative cases treated with conventional orthodontic method (Panels **a** and **b**) or with PBM (Panels **c** and **d**). Panel **a** Baseline (Day 0); LII is 8.80 mm. Panel **b**. Day 131; LII is 0.00 mm. Panel **c**. Baseline (Day 0); LII is 9.07 mm. Panel **d**. Day 50; LII is 0.00 mm

**Fig. 4:**
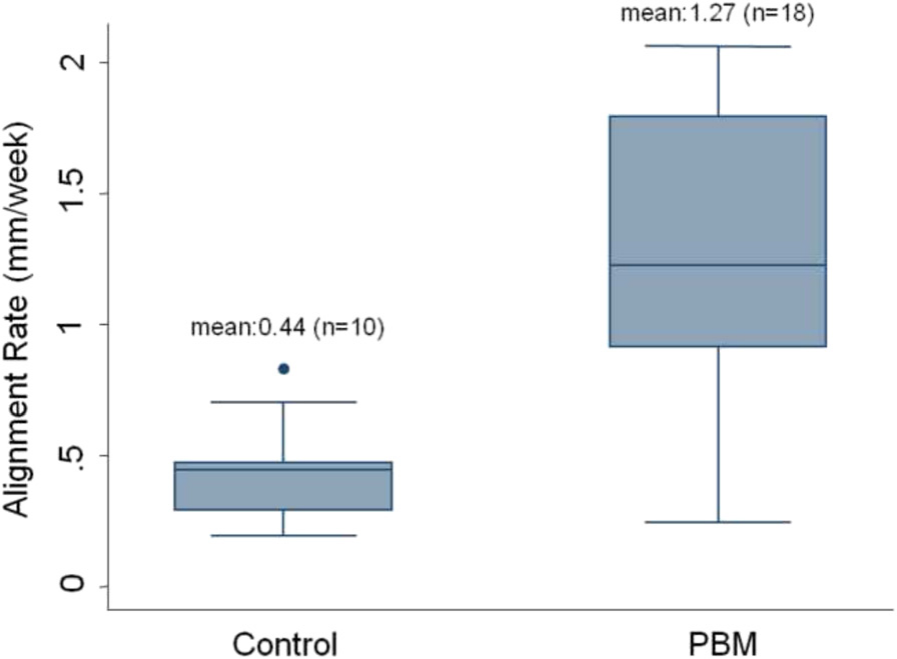
Boxplots showing alignment rates (mm/week) for control and PBM treated patients. Whiskers represent 1.5-times the interquartile ranges. Outliers are included. A statistically significant difference (*p* = 0.0002) in alignment rate was found between the two groups. The PBM group’s mean alignment rate was 1.27 (Interval of 1.01-1.53 at 95 % confidence), compared to a control rate of 0.44 (Interval of 0.30-0.59 at 95 % confidence) with a comparison group of 10 control arches and 18 PBM-treated arches

Representative intraoral photographs of control and PBM treated patients are shown in Fig. 3. The PBM-treated group was found to have aligned at a rate of 1.27 mm/week compared to 0.44 mm/week for the control group (Fig. 4) (*p* = 0.0002, Mann-Whitney U). The duration required for anterior tooth alignment was significantly less for the PBM than for the control group (*p* = 0.0053), with mean alignment times of 48 and 104 days, respectively (Table 2). There was no statistically significant difference in initial LII between the two groups (Table 3). However, the maximum starting LII was 8.80 mm for control and 14.58 mm for PBM.

**Table 2:** Treatment time to alignment in days for each group

		# of Arches	Mean (SD)	Median	Min–Max	*p*-value*
Group						
	Control	10	104 (55)	102	42–204	0.0053
	PBM	18	48 (39)	37	17–164	

*p-value based on the two-tailed Mann-Whitney U. A *p*-value cutoff of 0.05 is used to determine significance

**Table 3:** LII measurements in millimeters (mm) at T0 and T1 for each group

		# of Arches	Mean (SD)	Median	Min–Max	*p*-value*
T0						
	Control Total	10	5.77 (1.57)	5.80	3.70–8.80	0.1572
	Upper	3	5.20 (1.30)	5.90	3.70–6.00	
	Lower	7	6.01 (1.70)	5.70	4.20–3.70	
	PBM Total	18	7.27 (2.99)	6.32	3.72–14.58	0.1572
	Upper	10	7.91 (3.41)	6.59	4.36–14.58	
	Lower	8	6.48 (2.33)	6.06	3.72–10.60	
T1						
	Control Total	10	0.33 (0.38)	0.25	0.00–1.00	0.3293
	Upper	3	0.33 (0.58)	0.00	0.00–1.00	
	Lower	7	0.33 (0.33)	0.50	0.00–0.80	
	PBM Total	18	0.47 (0.41)	0.58	0.00–1.17	0.3293
	Upper	10	0.44 (0.39)	0.60	0.00–0.99	
	Lower	8	0.50 (0.46)	0.55	0.00–1.17	

*p-value based on two-tailed Mann-Whitney U. A *p*-value cutoff of 0.05 is used to determine significance

The Cox proportional hazard model has been used to examine difference in orthodontic tooth movement rates[36, 37]. Our results are presented over five models (Table 4). The first three models are a baseline set of controls covering all available demographic and clinical characteristics. Model 4 is the fully specified model, including PBM. To address any concerns surrounding potential model over-fitting[38], the three independent variables with the lowest *p*-values were removed from Model 4. The crowding resolution ratios were substantively unchanged by this (from 4.661 to 4.397, *p* < 0.01). Therefore, our findings demonstrate that in the current dataset alignment rate is not influenced by any of these parameters, except for age –older patients appear to reach arch alignment later than younger patients. Initial crowding exhibited evidence of marginal significance, with higher LII associated with longer treatment (0.10 > *p* > 0.05 in models 4 and 5). These findings are consistent with previous findings[38, 39].

**Table 4:** Cox proportional hazard models

	Model 1	Model 2	Model 3	Model 4	Model 5
Age	0.886	0.837	0.819	0.657*	0.672*
Female	1.071	1.288	1.164	1.156	
Caucasian	1.004	1.144	1.245	1.477	
Mandible		0.480+	0.756	0.829	
Crowding (LII - mm)			0.544*	0.549+	0.513+
PBM				4.661**	4.397**
Arches	28	28	28	28	28
Failures	28	28	28	28	28
N	37	37	37	37	37
dF	3	4	5	6	3
chi2	0.813	3.549	9.315	17.725	16.786

+ *p* < 0.010, * *p* < 0.05 ** *p* < 0.01

**Fig. 5:**
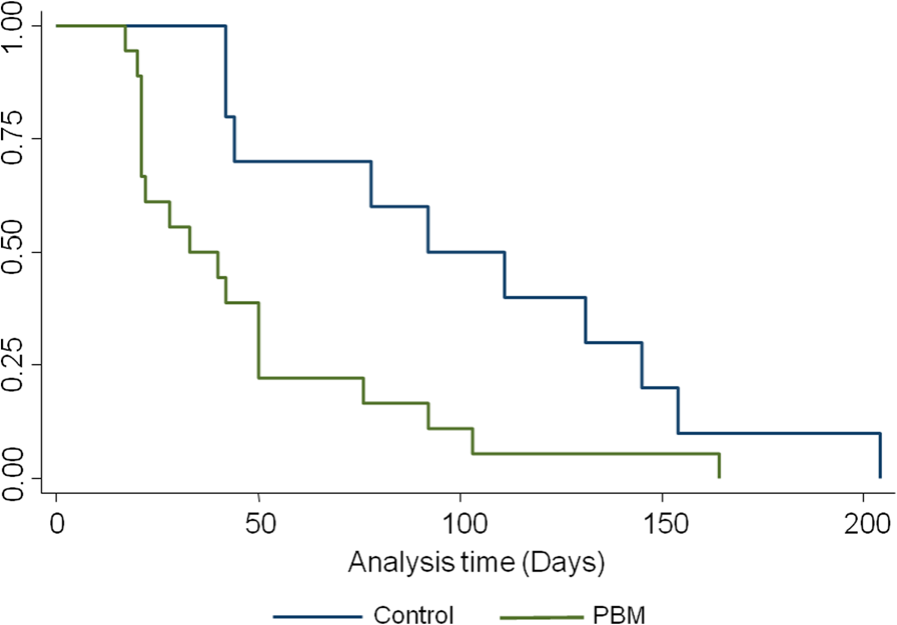
Kaplan-Meier survival curves for the two groups used in the study. The *y*-axis gives the proportion of patients still in treatment (not aligned) over time (days on *x*-axis). By drawing a line perpendicular to the *x*-axis at a given time value, the proportion of patients not completed for each group is determined from the corresponding *y*-axis. There is clear separation occurring as early as 20 days. This separation is maintained throughout the duration of the study

Only when PBM is introduced is a significant difference in alignment rate observed between groups. The difference in treatment time of the two groups shows a clear separation as early as 20 days, which was maintained throughout the duration of the study (Fig. 5).

Based on these results, we reject the null hypothesis that there would be no difference in the rate of anterior orthodontic alignment between the control and PBM groups.

Intra-examiner reliability was strong, with an ICC of over 0.96. Even when comparing only non-zero contact points, an ICC of over 0.94 was obtained. Values greater than 0.75 are typically regarded as excellent[40]. A *p*-value of 0.87 was obtained using the Mann-Whitney U to test the median difference between the first and second measurement sets.

## Discussion

This study used anterior tooth alignment to assess tooth movement and required treatment time. Adjunctive intraoral IR PBM appeared to significantly increase the rate of tooth movement, decreasing alignment time. PBM-treated arches exhibited a mean alignment rate of 1.27 mm/week and alignment time of 48 days, versus the control’s 0.44 mm/week and 104 days (Fig. 4, Table 2). Patient PBM compliance was high, averaging 93 %.

Several previous laboratory studies have evaluated the effect of PBM on bone remodeling in animal subjects. Many have shown that PBM accelerated these processes[23]. The methods in this study differ slightly from those seen in previous LLLT or PBM studies[24, 25]. These studies recorded canine tooth retraction, which entails bodily movement, where ours assessed crowding resolution -a product of rotation and tipping. They also used hand-held lasers and different IR treatment protocols - most notably, fewer treatments than the current study.

The use of hand-held lasers by a skilled operator is both time consuming and requires additional orthodontic visits. For example, the Doshi-Meta and Bhad-Patil protocols required 100 s of laser treatment per tooth[2]. For 12 anterior teeth, this requires an additional 20 min of the operator’s time per patient. This may not be practical in the typical clinic, where there is not always time for extra or longer appointments. The self-treatments PBM device used in this study pre-empts the need for more chair time.

The device used here is also an improvement over the extraoral device used in our previous study[31]*.* The extraoral unit had both a higher power density (60 mW/cm^2^) and longer treatment times. The intraoral device allows direct contact with periodontal tissues and alveolar bone, which minimizes the absorption of light by the soft tissues of the cheeks. Despite applying a lower energy density, use of the intraoral device demonstrated a similar increase in alignment rate to the extraoral device, accelerating tooth alignment over 2-fold compared to control. This suggests that applying NIR light directly to the surface of the alveolar mucosa allows for lower energy density to achieve a similar effect, as soft tissue and blood have been shown to absorb as much as 80–90 % of incoming irradiation[41].

The effectiveness of intraoral treatment may also be explained by the biphasic nature of the PBM response[42]. There is evidence that a dose threshold exists which must be overcome for PBM to have any biological effects[43]. If so, the power density must be high enough to reach the depth of the cellular components responsible for tooth movement[44]. Excessive dosages, though, may be detrimental to the effect of PBM[45]. Goulart et al.[46] observed that high doses of laser therapy reduced the rate of tooth movement in dogs, when the opposite was seen at lower doses.

New studies are needed to determine the effects of power density and application time on the rate of tooth alignment. Testing these variables on large and varied treatment groups may provide insight into the threshold level and optimal dosage most applicable to the general population.

### Limitations

We have taken measures to prevent bias when designing this study. First, we clearly and extensively communicated with patients and their legal representatives. This phase included oral and written consent – an independent review board approved all consent documents. Second, data was collected from study models by an independent, blinded investigator. Third, an independent statistician analyzed the data. Separate and blinded treatment of data serves to reduce investigator bias.

This trial also asked whether the orthodontic benefits of PBM could be combined with practical ease of use. However, it was designed as preliminary, and as such comes with shortcomings: Group assignments were not randomized (the control group was enrolled in full prior to PBM enrolment), there was no sham control, patients were mixed between conventional and self-ligating brackets, the study duration was only until alignment, the sample size was small and the arch distribution in each group was not perfectly uniform.

Because groups were enrolled sequentially, most members of the PBM group were bonded with 0.018-in slot conventionally-ligated (CL) Ormco Mini-Diamond brackets, while the control group and the last few PBM patients were bonded with 0.018-in slot self-ligating (SL) SPEED brackets[47]. SL brackets provide less friction, presumably allowing teeth to move faster. This suggests that a faster rate of tooth movement would be observed in the control group, had PBM had no effect. Nevertheless, many studies, including a recent systematic review, have found no difference in anterior crowding resolution time between CL bracket cases and SL cases[36, 48–51].

There is evidence that arch-wire type and wire sequencing has more bearing on tooth movement rate than bracket ligation type[52]. Patients in both groups progressed through alignment with NiTi arch-wires from 0.014-in through to 0.018-in (Ormco). Arch-wires were changed in the same manner for every patient – only when they were passively engaged in all brackets slots, as determined by the investigating orthodontist. A future study should use a consistent bracket ligation type across treatment groups and follow treatment groups until completion of orthodontic treatment.

To best address the limitations of this study, we conducted an extensive statistical analysis using Cox model multiple regression. The Cox model estimates the occurrence of an event – tooth alignment – as a function of independent variables, as shown in Table 4. Its main advantage is in controlling for multiple independent variables. Our model shows a significantly faster rate of tooth alignment in the PBM group versus control when controlling for age, gender, ethnicity, degree of initial crowding and treatment arch (mandible or maxilla).

## Conclusions

Under the limitations of this pilot study, it could be suggested that:

- Intraoral photobiomodulation produced statistically significant changes in the rate of tooth movement during the alignment phase of orthodontic treatment when controlling for demographic factors, degree of crowding and whether the movement was in the maxillary or mandibular arch.
- Overall treatment time was significantly reduced in subjects treated with intraoral PBM compared to control, with treatment groups achieving anterior tooth alignment in an average of 48 days, versus a control average of 104 days.
- Considering the limitations of this study, there is need for a larger, randomized, sham-control clinical trial to further investigate the effects of daily, intraoral photobiomodulation on orthodontic tooth movement

## Availability of supporting data

All supporting data are included as additional files, upon request.

## Abbreviations

ATP: Adenosine Triphosphate
CL: Conventionally-Ligated
ICC: Interclass Correlation Coefficient
IRB: Institutional Review Board
LED: Light Emitting Diode
LII: Little’s Irregularity Index
NIR: Near Infrared
NS: Not Significant
PBM: Photobiomodulation
RAP: Regional Accelerating Phenomenon
SD: Standard Deviation
SL: Self-Ligating
T.S: Timothy Shaughnessy (Investigating Orthodontist)
